# Dietary fat quality indices and risk of pre-diabetes and type 2 diabetes mellitus: Tehran Lipid and Glucose Study

**DOI:** 10.1017/S1368980024001216

**Published:** 2024-12-09

**Authors:** Zahra Gaeini, Sevda Alvirdizadeh, Somayeh Hosseinpour-Niazi, Parvin Mirmiran, Fereidoun Azizi

**Affiliations:** 1 Nutrition and Endocrine Research Center, Research Institute for Endocrine Sciences, Shahid Beheshti University of Medical Sciences, Tehran, Iran; 2 Department of Clinical Nutrition and Dietetics, Faculty of Nutrition and Food Technology, Nutrition and Endocrine Research Center, Research Institute for Endocrine Sciences, Shahid Beheshti University of Medical Sciences, Tehran, Iran; 3 Endocrine Research Center, Research Institute for Endocrine Sciences, Shahid Beheshti University of Medical Sciences, Tehran, Iran

**Keywords:** Fat quality indices, Pre-diabetes, Type 2 diabetes mellitus

## Abstract

**Objective:**

To assess associations between dietary fat quality indices and risk of pre-diabetes and type 2 diabetes mellitus (T2DM) among Iranian adults.

**Design:**

Daily intakes of fatty acids were estimated using a validated FFQ with 168 food items. Adjusted hazard ratios (HR) and 95 % CI for pre-diabetes and T2DM were calculated across tertile categories of dietary fat quality indices including the atherogenic index, thrombogenic index, health-promoting index, ratio of PUFA to SFA (PUFA:SFA) and ratio of hypo- and hypercholesterolaemia (h:H).

**Setting:**

Tehran Lipid and Glucose Study.

**Participants:**

Iranian men and women.

**Results:**

The mean (sd) age of the 2042 pre-diabetes-free participants in pre-diabetes analysis was 38·84 (12·97), and 55·2 % were women. In T2DM analysis, the mean (sd) age of the 2295 T2DM-free participants was 40·06 (13·42), and 54·6 % of them were women. In the crude model, the PUFA:SFA ratio was positively associated with T2DM incidence (HR = 1·43; 95 % CI 1·04, 1·98). However, after adjustment for confounding variables, there were no significant associations between dietary fat quality indices and risk of pre-diabetes and T2DM.

**Conclusions:**

We found no significant association between fat quality indices and risk of pre-diabetes and T2DM. Further prospective and clinical trial studies are needed to clarify the issue.

Type 2 diabetes mellitus (T2DM) is a worldwide health problem that is defined by impaired metabolism of carbohydrates, proteins and fats owing to erratic insulin production, insulin resistance or both^(1)^. ‘Pre-diabetes’ refers to an intermediate stage between normal glucose tolerance and overt T2DM. Individuals with pre-diabetes have a higher tendency to develop T2DM^(2)^. The association between the amount of dietary fat intake and the risk of T2DM has been investigated in numerous studies; however, the results were inconsistent, and the conclusions were underpowered. Three cohort studies have shown an independent association between total fat intake and the incidence of T2DM^(3–5)^. However, some other cohort studies didn’t show the association^(6–9)^. One recent meta-analysis also reported no significant association between total fat intake and T2DM incidence^(10)^.

Based on previous studies, a consensus has not yet been reached regarding the impact of the quality of dietary fats on the development of metabolic disorders. Recent studies suggested that to consider the balance between the positive and negative effects of dietary fats on the risk of diseases, investigation of dietary fat quality indices (FQI) might be helpful^(11,12)^. During the last decades, some studies proposed different dietary FQI, including the atherogenic index (AI)^(13)^, thrombogenic index (TI)^(13)^, health-promoting index (HPI)^(14)^, ratio of PUFA to SFA (PUFA:SFA) and ratio of hypo- to hypercholesterolemic fatty acids (h:H)^(15)^, which might have an effect on metabolic abnormalities. To the best of our knowledge, little is known about the association between individual FQI and the risk of T2DM or pre-diabetes. In a previous related study, AI was found to be positively associated with pre-diabetes incidence, while the *n*-3:*n*-6 and h:H ratios were negatively associated^(16)^. Despite this, the association between FQI and other metabolic outcomes has been investigated in a number of studies^(17–19)^, and they have shown conflicting results.

Regarding the very limited number of previous cohort studies that assessed dietary FQI and their association with the risk of T2DM and pre-diabetes, the present study aimed to investigate the association between dietary FQI and risk of pre-diabetes and T2DM among an Asian adult population.

## Methods

### Study population and measurements

**Figure 1.**
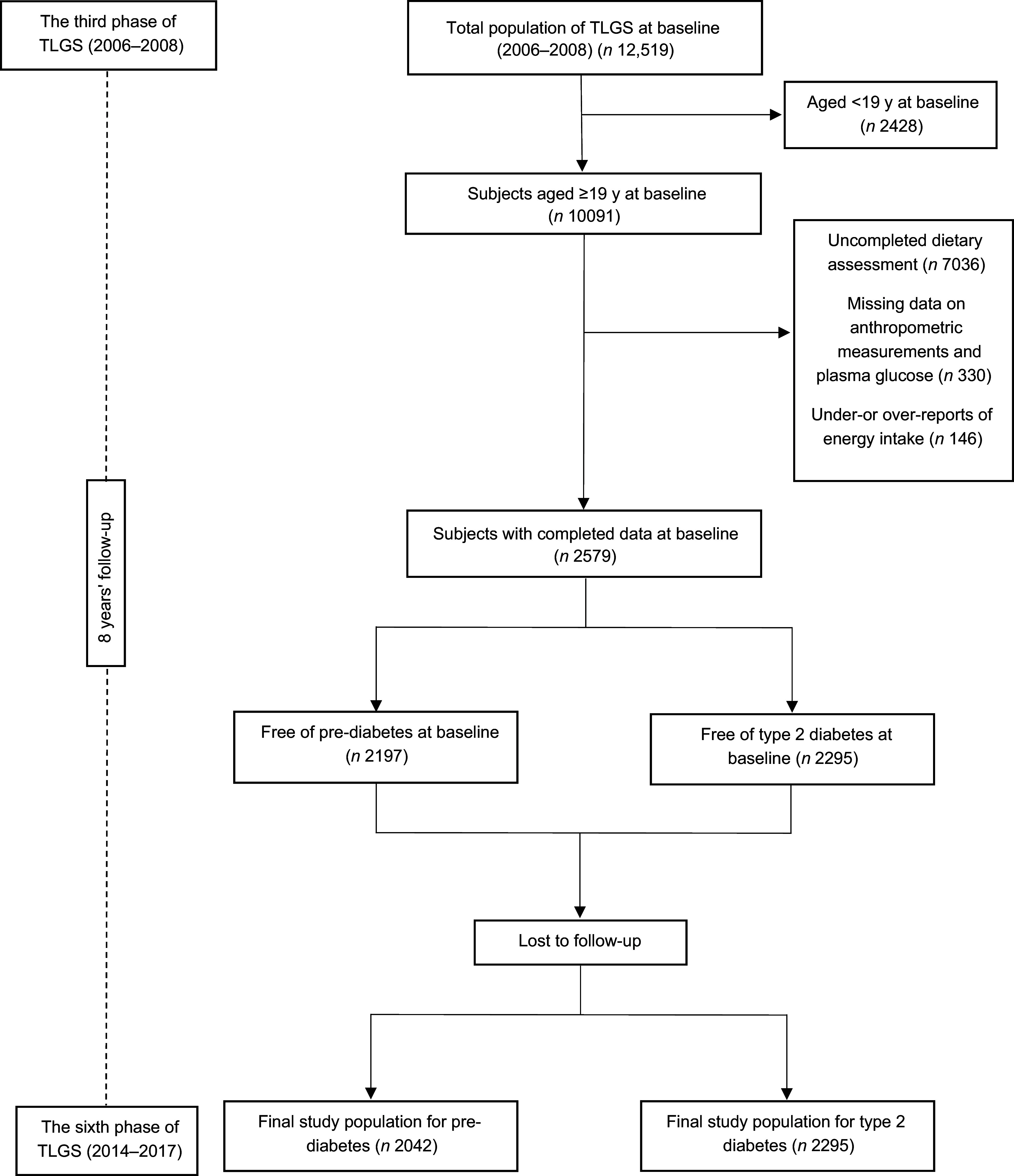
Flow chart of the study.

The present study was conducted within the framework of the Tehran Lipid and Glucose Study (TLGS), an ongoing prospective study initiated in 1998 to evaluate non-communicable disease risk factors among 15 005 Tehranian adults aged ≥ 3 years^(20)^. In the third phase of the TLGS (2006–2008), there were 10 091 adults aged ≥ 19 years with a complete medical history and physical examination data. Among those, we excluded participants who had uncompleted dietary data (*n* 7036), participants with missing data on anthropometric measurements and plasma glucose (*n* 330), participants with under- or over-reports of energy intake (< 800 kcal/d or > 4200 kcal/d, respectively) (*n* 146), participants with pre-diabetes (*n* 382) or T2DM (*n* 284) at baseline and participants who lost to follow-up. The final analyses were conducted on data of 2042 adults free of pre-diabetes and 2295 adults free of T2DM (Fig. 1). The eligible participants were followed up to the sixth phase of the TLGS (2014–2017). The median periods of follow-up were 7·37 and 8·47 years from baseline, for pre-diabetes and T2DM, respectively.

### Anthropometric and demographic assessments

Data on anthropometric, demographic and biochemical variables were collected by trained interviewers of TLGS at baseline (2006–2008). The body weight and height of participants were measured to the nearest 100 g and 0·5 cm, using digital scales (Seca) and a tape metre, respectively. BMI was calculated as weight (kg) divided by the square of height (m^2^). Waist circumference (WC) was measured to the nearest of 0·1 cm, from midway between the lower border of the ribs and the iliac crest at the widest portion, using a soft measuring tape. To measure systolic and diastolic blood pressures, we used a standard mercury sphygmomanometer calibrated by the Iranian Institute of Standards and Industrial Research^(21)^. The blood pressure of participants was measured twice on the right arm with a 30-s interval between two measurements. The final blood pressure of participants was considered as the mean of the two measurements. The usual physical activity of participants was evaluated using the modifiable activity questionnaire (MAQ). Physical activity levels were expressed as metabolic equivalent minutes per week (MET-min/week) and categorised as low physical activity (scores ≤ 600 MET-min/week) and moderate and high physical activity (scores > 600 MET-min/week)^(22)^. The reliability and validity of the Persian version of the MAQ were previously investigated^(23)^.

### Biochemical measurements

A blood sample was taken after overnight fasting between 07.00 and 09.00 from all participants. All blood analysis was done at the research laboratory of the TLGS, using Pars Azmoon kits (Pars Azmoon Inc.) and a Selectra 2 auto-analyser (VitalScientific). Fasting serum glucose and 2-h serum glucose were measured by the enzymatic colorimetric method using glucose oxidase. Serum TAG was measured by the enzymatic colorimetric method using glycerol phosphate oxidase. HDL-cholesterol was measured after precipitation of the apo B-containing lipoproteins with phosphotungstic acid. Both inter- and intra-assay CV at baseline and follow-up phase were less than 5 %.

### Dietary assessment

The dietary intake of participants over the previous year was assessed using a validated 168-item FFQ at baseline^(24)^. Participants were asked to designate their intake frequency for each food item consumed during the past year on a daily, weekly or monthly basis. The frequencies were then converted to daily intakes, and portion sizes, reported in household measures, were converted to grams^(25)^. Since the Iranian Food Composition Table is incomplete and has limited data on the nutrient content of raw foods and beverages, the United States Department of Agriculture (USDA) food composition table was used to obtain the amount of energy and macronutrients per gram of each type of food.

### Fat quality indices

AI is the sum of SFA divided by the sum of MUFA and *n*-3 and *n*-6 PUFA.

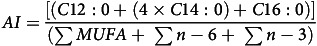




TI is attributed to the less anti-atherogenic effect of MUFA and *n*-6 PUFA, compared with the *n*-3 PUFA.

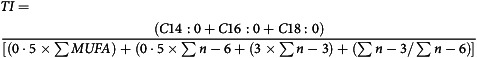




HPI is the sum of the unsaturated fats divided by the saturated fats.

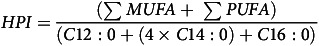




Ratio of PUFA and SFA is the sum of PUFA divided by the sum of SFA.

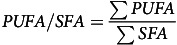




Ratio of hypo- and hypercholesterolaemia (h:H) is the sum of hypocholesterolemic PUFA divided by the sum of myristic acid and palmitic acid.

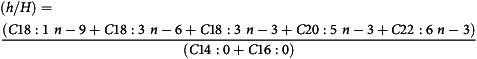




### Definition of terms and outcomes

Pre-diabetes was defined as impaired fasting glucose (fasting serum glucose levels of 5.56–6.95 mmol/l) or impaired glucose tolerance (2h-serum glucose values of 7.78–11.06 mmol/l). T2DM was defined as fasting serum glucose ≥ 7.00 mmol/l, 2h-serum glucose ≥ 11.11 mmol/l or self-reported taking of anti-diabetic medications^(26)^. Having at least one parent or sibling with T2DM is considered a positive family history of T2DM. The diabetes risk score (DRS) was calculated as follows: systolic blood pressure (mm Hg) < 120 (0 point), 120 < systolic blood pressure < 140 (3 points) and systolic blood pressure ≥ 140 (7 points); family history of diabetes (5 points); WC:height ratio: < 0·54 (0 point), 0·54–0·59 (6 points) and ≥ 0·59 (11 points); TAG:HDL-cholesterol ratio: < 3·5 (0 point) and ≥ 3·5 (3 points); fasting serum glucose (mmol/l): < 5 (0 point), 5–5.5 (12 points) and 5.56–6.89 (33 points)^(27)^.

### Statistical analyses

The incidence of pre-diabetes and T2DM over the follow-up period were considered as dichotomous variables (yes/no) in the models. Mean (sd) values and frequencies (%) of baseline characteristics of participants were compared according to the incidence of pre-diabetes and T2DM, using ANOVA and *χ* ^2^ test, respectively. Dietary FQI were categorised into tertiles. The first tertile of each category was considered as a reference.

Cox proportional hazard regression models with person-years as the underlying time metric were used to estimate hazard ratios (HR) and 95 % CI for the association between dietary FQI (AI, TI, HPI, PUFA:SFA and h:H) and risk of pre-diabetes and T2DM. The event date for cases was defined as the middle-time between the date of the follow-up visit at which pre-diabetes/T2DM was detected for the first time and the most recent follow-up visit preceding the diagnosis; the follow-up time was drawn from the difference between the calculated mid-time date and the date at which the subjects entered the study. The survival time was the interval between the first and the last observation dates for the censored and lost to follow-up subjects. The proportional hazard assumption of the multivariable Cox model was assessed using Schoenfeld’s global test of residuals. Sensitivity analysis was conducted after the exclusion of subjects with pre-diabetes at baseline in T2DM models. This approach was taken to assess if the results may have been influenced by the pre-diabetes subjects.

Potential confounders were determined using univariate analysis. Variables with P_E_ less than 0·2 in the univariate analyses, which were selected as confounders and adjusted in the Cox models, included sex (male/female), age (years), BMI (kg/m^2^), smoking (yes/no), physical activity level (low/moderate and high), DRS (continuous), socio-economic status (marital status, employment status and education level), total energy (kcal/d) and total fibre intake (g/d). Adjustment of DRS, as a continuous potential risk factor of T2DM, improved the stability of our models due to the limited number of outcomes.

All statistical analyses were performed using the Statistical Package for Social Science (version 20; IBM Corp.), with *P*-values < 0·05 being considered significant.

## Results

Baseline characteristics of participants and dietary FQI according to the status of pre-diabetes and T2DM are summarised in Table 1 and Table 2. During an average of 7·37 (interquartile range = 3·99–9·04) years of follow-up, the incidence rate of pre-diabetes was 40·2 %. Participants with pre-diabetes tended to be older and had higher WC, TAG/HDL-cholesterol and DRS compared with the participants with no pre-diabetes incidence. Also, participants with pre-diabetes had a significantly higher daily intake of total fibre and lower daily intakes of total fat (percentage from total energy), total SFA (g/d and percentage from total energy), total MUFA (percentage from total energy), lauric acid (C12:0), myristic acid (C14:0) and EPA (C20:5, *n*-3), compared with those with no pre-diabetes outcomes (*P*_for all_ < 0·05). Over a median of 8·47 (interquartile range = 6·33–9·41) years of follow-up, a total of 243 cases of T2DM were identified among 2295 participants (incidence rate = 10·6 %). Compared with the participants with no T2DM outcomes, participants with T2DM had higher WC, TAG/HDL-cholesterol and DRS. Furthermore, participants with T2DM had significantly lower daily intakes of total SFA (percentage from total energy), myristic acid (C14:0), erucic acid (C22:1) and total PUFA and higher daily intake of linoleic acid (C18:2, *n*-6) (*P*_for all_ < 0·05), compared with those without T2DM incidence. There was no significant difference in other baseline characteristics between the two groups. Moreover, there was no significant difference in any of the FQI between subjects with or without pre-diabetes and T2DM (Table 2).

**Table 1:** Baseline characteristics of participants according to incident pre-diabetes and T2DM

Baseline characteristics	Pre-diabetes ^+^		Pre-diabetes ^_^		T2DM ^+^		T2DM ^–^	
	(*n* 822)		(*n* 1220)		(*n* 243)		(*n* 2052)	
	Mean	sd	Mean	sd	Mean	sd	Mean	sd
Age, year	43·87	13·12	35·45	11·71*	47·35	13·05	39·20	13·20
	%		%		%		%	
Male, %	53·3		39·1		46·9		45·2	
Current smoker, %	10·2		8·9		8·8		9·5	
Low physical activity, %	36·8		39·8		40·4		38·6	

	Mean	sd	Mean	sd	Mean	sd	Mean	sd
BMI, kg/m^2^	27·87	4·54	25·79	4·54	30·25	4·85	26·58	4·60
Waist circumference, cm	92·76	11·27	85·20	12·70*	98·32	10·50	88·42	12·70*
TAG/HDL-cholesterol	4·09	2·94	3·05	2·38*	5·03	3·26	3·46	2·67*
Diabetes risk score	23·04	14·24	12·11	12·01*	32·82	15·55	17·28	14·71*
Total energy intake, kcal/d	2256	725·5	2277	701·5	2199	723·1	2269	713·9
Total fibre intake, g/d	38·45	21·82	36·61	18·87*	38·71	21·22	37·40	20·75
Dietary fatty acids intake								
Total fat, g/d	76·51	30·76	80·60	31·19	75·29	33·38	78·90	31·14
Total fat, % energy	30·49	7·00	31·85	6·93*	30·69	7·78	31·27	6·98
Total SFA, g/d	25·22	10·76	27·75	18·70*	24·33	10·21	26·48	11·72
Total SFA, % energy	10·07	2·75	10·95	6·17*	10·00	2·91	10·48	3·00*
Lauric acid (C12:0), g/d	0·83	0·66	0·94	0·77*	0·79	0·54	0·90	0·76
Myristic acid (C14:0), g/d	2·05	1·08	2·29	1·33*	1·98	0·97	2·19	1·26*
Palmitic acid (C16:0), g/d	11·95	5·40	12·66	5·50	11·68	5·73	12·36	5·54
Stearic acid (C18:0), g/d	4·85	2·12	5·25	2·27	4·67	2·14	5·09	2·23
Total MUFA, g/d	26·36	11·10	27·95	11·32	26·26	12·76	27·27	11·26
Total MUFA, % energy	10·53	2·83	11·06	2·76*	10·69	3·19	10·83	2·80
Myristoleic acid (C14:1), g/d	0·03	0·03	0·03	0·03	0·03	0·02	0·03	0·03
Palmitoleic acid (C16:1), g/d	0·67	0·42	0·73	0·38	0·64	0·31	0·71	0·40
Oleic acid (C18:1), g/d	14·15	7·52	15·19	7·19	13·87	7·58	14·73	7·35
Gadoleic acid (C20:1), g/d	0·20	0·43	0·20	0·21	0·18	0·13	0·20	0·32
Erucic acid (C22:1), g/d	0·006	0·006	0·006	0·006	0·005	0·004	0·006	0·006*
Nervonic acid (C24:1), g/d	0·001	0·0009	0·001	0·001	0·0009	0·0009	0·0011	0·001
Total PUFA, g/d	16·10	8·11	16·63	7·75	16·08	9·76	16·40	7·80*
Total PUFA, % energy	6·42	2·39	6·59	2·24	6·49	2·69	6·52	2·29
Linoleic acid (C18:2, *n*-6), g/d	6·30	4·75	6·52	4·18	6·78	7·52	6·40	4·37*
*α*-Linolenic acid (C18:3, *n*-3), g/d	0·41	0·39	0·42	0·31	0·39	0·36	0·41	0·34
*γ*-Linolenic acid (C18:3, *n*-6), g/d	0·009	0·009	0·01	0·008	0·009	0·007	0·01	0·008
EDA (C20:2, *n*-6), g/d	0·008	0·006	0·008	0·005	0·007	0·005	0·008	0·005
AA (C20:4, *n*-6), g/d	0·17	0·40	0·16	0·19	0·15	0·12	0·17	0·29
EPA (C20:5, *n*-3), g/d	0·03	0·12	0·03	0·04*	0·03	0·03	0·03	0·08
DHA (C22:6, *n*-3), g/d	0·11	0·37	0·09	0·15	0·09	0·10	0·10	0·26

T2DM, type 2 diabetes mellitus; EDA, eicosadienoic acid; AA, arachidonic acid.

Data are mean (sd) unless stated otherwise.

* *P*-value < 0·005. Independent *t* test and *χ* ^2^ test were used for continuous and dichotomous variables, respectively. Mean (sd) and frequencies were compared between pre-diabetes and healthy subjects and type 2 diabetes patients and healthy subjects.

**Table 2:** Dietary fat quality indices according to incident pre-diabetes and T2DM

Fat quality indices	Pre-diabetes^+^		Pre-diabetes^_^		T2DM^+^		T2DM^–^	
	(*n* 822)		(*n* 1220)		(*n* 243)		(*n* 2052)	
	Mean	sd	Mean	sd	Mean	sd	Mean	sd
Atherogenic index	8·54	5·09	8·76	4·33	11·80	7·32	11·40	4·38
Thrombogenic index	1·65	0·71	1·65	0·80	12·84	5·71	12·61	4·69
Health-promoting index	1·12	0·44	1·11	0·43	0·69	0·47	0·65	0·36
Ratio of PUFA and SFA	0·35	0·22	0·34	0·20	0·37	0·26	0·34	0·20
Ratio of hypo- and hypercholesterolaemia	0·84	0·49	0·84	0·44	0·88	0·59	0·83	0·45

T2DM, type 2 diabetes mellitus.

Data are mean (sd); independent *t* test was used.

The HR and 95 % CI of pre-diabetes and T2DM across tertile categories of dietary FQI are shown in Table 3 and Table 4. There was a positive association between the ratio of dietary PUFA to SFA and risk of T2DM in the crude model (HR = 1·43; 95 % CI 1·04, 1·98; *P*_for trend_ = 0·030); however, the association has not remained significant in the adjusted models. There were no significant associations between dietary FQI (AI, TI, HPI, PUFA:SFA, h:H) and pre-diabetes and T2DM risk in the adjusted models. Sensitivity analysis was conducted by excluding the subjects with pre-diabetes at baseline from the T2DM models to assess if the associations between dietary FQI and risk of T2DM have been influenced by the pre-diabetes status of the subjects. The results of sensitivity analyses have shown no changes in the direction and significance of the associations when the subjects with pre-diabetes were excluded (Table 4). Moreover, when the analyses were conducted without BMI as a confounder, there was no significant association found between dietary FQI and pre-diabetes and T2DM risk in that model (Table 3 and Table 4).

**Table 3:** Risk of pre-diabetes according to tertiles of dietary fat quality indices

	Tertiles					*P*_for trend_
	T1	T2		T3		
Atherogenic index						
Range	< 6·50	6·51–9·18		> 9·19		
HR (95 % CI) crude model	1·00	0·94	0·79, 1·11	0·91	0·77, 1·08	0·296
HR (95 % CI) model 1	1·00	0·99	0·83, 1·17	1·05	0·88, 1·25	0·695
HR (95 % CI) model 2	1·00	1·00	0·83, 1·19	1·07	0·88, 1·29	0·640
HR (95 % CI) model 3	1·00	0·99	0·77, 1·28	1·06	0·79, 1·41	0·717
Thrombogenic index						
Range	< 1·33	1·34–1·73		> 1·74		
HR (95 % CI) crude model	1·00	1·06	0·90, 1·26	0·96	0·80, 1·14	0·618
HR (95 % CI) model 1	1·00	0·98	0·82, 1·16	0·84	0·71, 1·01	0·065
HR (95 % CI) model 2	1·00	0·98	0·82, 1·16	0·85	0·71, 1·01	0·067
HR (95 % CI) model 3	1·00	0·88	0·68, 1·13	0·82	0·64, 1·05	0·122
Health-promoting index						
Range	< 0·90	0·91–1·21		> 1·22		
HR (95 % CI) crude model	1·00	1·00	0·84, 1·18	0·99	0·83, 1·17	0·894
HR (95 % CI) model 1	1·00	1·07	0·90, 1·27	1·13	0·95, 1·34	0·201
HR (95 % CI) model 2	1·00	1·07	0·90, 1·27	1·13	0·94, 1·33	0·205
HR (95 % CI) model 3	1·00	1·09	0·85, 1·39	1·09	0·85, 1·41	0·488
Ratio of PUFA and SFA						
Range	< 0·23	0·24–0·37		> 0·38		
HR (95 % CI) crude model	1·00	1·11	0·94, 1·32	1·13	0·95, 1·34	0·170
HR (95 % CI) model 1	1·00	1·15	0·97, 1·37	1·19	0·98, 1·40	0·061
HR (95 % CI) model 2	1·00	1·16	0·97, 1·38	1·18	0·99, 1·41	0·060
HR (95 % CI) model 3	1·00	1·21	0·94, 1·55	1·22	0·94, 1·58	0·134
Ratio of hypo- and hypercholesterolaemia						
Range	< 0·61	0·62–0·93		> 0·94		
HR (95 % CI) crude model	1·00	1·02	0·86, 1·21	0·99	0·84, 1·18	0·935
HR (95 % CI) model 1	1·00	1·12	0·94, 1·32	1·09	0·92, 1·30	0·360
HR (95 % CI) model 2	1·00	1·11	0·94, 1·32	1·08	0·91, 1·29	0·395
HR (95 % CI) model 3	1·00	1·10	0·86, 1·40	1·08	0·83, 1·41	0·528

Data are hazard ratio (95 % CI); proportional hazard Cox regression was used. HR, hazard ratio.

Model 1 adjusted for sex, age, BMI, smoking, physical activity, diabetes risk score and socio-economic status.

Model 2 additionally adjusted for total energy (kcal/d) and total fibre (g/d).

Model 3 adjusted for all mentioned confounders except BMI.

**Table 4:** Risk of type 2 diabetes according to tertiles of dietary fat quality indices

	Tertiles							
	T1	T2		T3		Sensitivity analysis (T3)		*P*_for trend_
Atherogenic index								
Range	< 9·46	9·47–11·85		> 11·86				
HR (95 % CI) crude model	1·00	0·85	0·62, 1·16	0·92	0·67, 1·25	1·09	0·71, 1·66	0·587
HR (95 % CI) model 1	1·00	0·92	0·67, 1·26	0·98	0·72, 1·34	1·07	0·70, 1·63	0·843
HR (95 % CI) model 2	1·00	0·95	0·68, 1·32	1·04	0·72, 1·52	1·29	0·78, 2·12	0·870
HR (95 % CI) model 3	1·00	1·09	0·65, 1·81	0·93	0·49, 1·78	1·40	0·84, 2·31	0·881
Thrombogenic index								
Range	< 10·73	10·74–13·11		> 13·12				
HR (95 % CI) crude model	1·00	1·06	0·77, 1·45	1·00	0·73, 1·37	1·07	0·69, 1·65	0·994
HR (95 % CI) model 1	1·00	1·04	0·76, 1·43	1·08	0·79, 1·49	1·08	0·70, 1·68	0·627
HR (95 % CI) model 2	1·00	1·09	0·78, 1·51	1·19	0·82, 1·71	1·32	0·80, 2·17	0·323
HR (95 % CI) model 3	1·00	1·28	0·75, 2·19	1·49	0·82, 2·71	1·41	0·86, 2·32	0·186
Health-promoting index								
Range	< 0·47	0·48–0·72		> 0·73				
HR (95 % CI) crude model	1·00	1·21	0·88, 1·66	1·24	0·90, 1·71	1·43	0·92, 2·23	0·188
HR (95 % CI) model 1	1·00	1·06	0·77, 1·47	1·19	0·86, 1·64	1·32	0·85, 2·06	0·380
HR (95 % CI) model 2	1·00	1·05	0·76, 1·45	1·16	0·84, 1·61	1·22	0·78, 1·92	0·484
HR (95 % CI) model 3	1·00	0·94	0·57, 1·57	0·90	0·53, 1·52	1·22	0·78, 1·81	0·694
Ratio of PUFA and SFA								
Range	< 0·23	0·24–0·37		> 0·38				
HR (95 % CI) crude model	1·00	1·31	0·94, 1·81	1·43	1·04, 1·98	1·48	0·95, 2·31	0·030
HR (95 % CI) model 1	1·00	1·13	0·81, 1·57	1·27	0·91, 1·75	1·30	0·84, 2·03	0·233
HR (95 % CI) model 2	1·00	1·12	0·81, 1·56	1·23	0·88, 1·72	1·19	0·76, 1·87	0·254
HR (95 % CI) model 3	1·00	0·87	0·53, 1·44	0·81	0·47, 1·39	1·18	0·76, 1·88	0·452
Ratio of hypo- and hypercholesterolaemia								
Range	< 0·61	0·62–0·93		> 0·94				
HR (95 % CI) crude model	1·00	1·18	0·85, 1·62	1·23	0·89, 1·69	1·40	0·90, 2·17	0·209
HR (95 % CI) model 1	1·00	1·10	0·80, 1·52	1·18	0·85, 1·63	1·36	0·87, 2·11	0·423
HR (95 % CI) model 2	1·00	1·09	0·79, 1·51	1·15	0·83, 1·60	1·26	0·81, 1·97	0·519
HR (95 % CI) model 3	1·00	0·99	0·60, 1·62	0·91	0·54, 1·54	1·21	0·78, 1·89	0·597

Data are hazard ratio (95 % CI); proportional hazard Cox regression was used. HR, hazard ratio.

Model 1 adjusted for sex, age, BMI, smoking, physical activity, diabetes risk score and socio-economic status.

Model 2 additionally adjusted for total energy (kcal/d) and total fibre (g/d).

Model 3 adjusted for all mentioned confounders except BMI.

## Discussion

In this large, prospective cohort study, we investigated the longitudinal association between dietary FQI including AI, TI, HPI, PUFA:SFA and h:H ratios and incidence of pre-diabetes and T2DM. The results from our data analysis did not show any significant associations between dietary FQI and risk of pre-diabetes and T2DM.

To the best of our knowledge, there is only one cross-sectional study that investigated the association between individual FQI and risk of pre-diabetes. This study, which was conducted among 150 subjects with normal fasting blood glucose and 147 pre-diabetic subjects, reported a positive association between AI and pre-diabetes (OR = 6·68; 95 % CI 2·57, 17·34) and a negative association between h:H ratio and pre-diabetes (OR = 0·20; 95 % CI 0·07, 0·52)^(16)^. They had shown no significant association between TI or PUFA:SFA ratio and pre-diabetes^(16)^. Unlike the aforementioned study, in the present study, we found a borderline inverse association for TI and a borderline positive association for PUFA:SFA in relation to pre-diabetes in fully adjusted models. Regarding the TI formulation, these two associations are in line with each other, indicating a borderline inverse association between dietary SFA and the risk of pre-diabetes. It may due to the fact that the main dietary source of SFA in the Iranian population is dairy products, which was inversely associated with the risk of pre-diabetes in previous cohort studies^(28,29)^. However, the controversies observed between the results may be due to the differences in study designs or sample sizes of the studies. As far as we know, there is no other study that investigated the long-term association between dietary FQI and the risk of pre-diabetes. Another cross-sectional study that investigated the relationship between dietary FQI and gestational diabetes mellitus revealed a positive association between TI and gestational diabetes mellitus (OR = 2·66; 95 % CI 1·34, 5·29) and a negative association between h:H ratio and gestational diabetes mellitus (OR = 0·41; 95 % CI 0·22, 0·77)^(30)^. However, their results showed no significant association between AI or PUFA:SFA and gestational diabetes mellitus, which is similar to our results^(30)^.

On the other hand, dietary FQI have been investigated in relation to some other metabolic disorders including CVD, general obesity and abdominal obesity, in a number of previous studies. No significant association was found between PUFA:SFA, as an index of dietary fat quality, and risk of CVD in a prospective cohort study (HR = 0·94; 95 % CI 0·61, 1·47 for the highest *v*. the lowest tertile, *P*_for trend_ = 0·884)^(31)^. General obesity and overweight were positively associated with TI (OR = 4·14; 95 % CI 1·78, 9·66; *P*_for trend_ = 0·01) and inversely associated with *n*-3:*n*-6 ratio (OR = 0·63; 95 % CI 0·24, 1·63; *P*_for trend_ = 0·005). Also, AI was found to be positively associated with abdominal obesity (OR = 1·24; 95 % CI 0·56, 2·74; *P*_for trend_ = 0·01)^(19)^. Furthermore, the association between FQI with AI of plasma was investigated in obese and non-obese individuals, where a positive correlation between AI, TI, SFA, PUFA, MUFA and *n*-6:*n*-3 ratio with AI of plasma and a negative correlation between h:H with AI of plasma in both groups were observed^(18)^.

Separate assessments of the independent association between individual fatty acids and the incidence of T2DM have not reached an agreement. The 2015–2020 Dietary Guidelines for Americans removed the limitation on total fat intake and placed emphasis on the quality of dietary fats within the context of a healthy dietary pattern and recommended reducing SFA intake to less than 10 % of the total calories^(32)^. SFA such as myristic and palmitic acids have been found to induce insulin resistance in animals^(33)^. Metabolic response to SFA can be related to the induction of serine–phosphorylation through activating specific serine kinases, which is the cause of the decrease in insulin-regulated GLUT-4 activity and less glucose uptake^(34)^. SFA can also impair insulin sensitivity by altering the membrane lipid composition, which results in the disorientation of membrane GLUT molecules^(35)^. However, available evidences supporting this recommendation to replace SFA and *trans*-fatty acids with PUFA and/or MUFA are inconsistent. One case–control study reported a positive association between intake of total SFA, myristic acid and palmitic acid and a negative association between *n*-3 PUFA, EPA, DHA and arachidonic acids intake and pre-diabetes^(16)^. Two systematic literature reviews suggested that *n*-6 PUFA (linoleic acid), but not *n*-3 PUFA, was inversely associated with the risk of T2DM and insulin resistance^(36,37)^. However, a recent meta-analysis study indicated no significant association between dietary total SFA and risk of T2DM^(38)^. Another meta-analysis study, which investigated the role of dietary fat type and quantity, suggested that replacing carbohydrates with any fat had no effect on fasting glucose^(39)^. One systematic review and meta-analysis study reported a neutral association between consumption of butter, as a major source of SFA, and T2DM^(40)^. Several other meta-analysis studies investigating the associations of high *v*. low-fat diets and fatty acid intakes with the incidence of T2DM^(41–45)^ did not support guidelines recommending increased intake of MUFA^(46)^, total *n*-3 PUFA^(47)^ or long-chain *n*-3 PUFA^(46)^ or lower intake of SFA^(48)^ to prevent T2DM.

The strengths of the present study include the prospective design, dietary assessment using a valid FFQ, detailed data on potential confounders and laboratory assessments for pre-diabetes and T2DM diagnosis. Using FQI instead of separate dietary intakes of fatty acids in this study to investigate the relationship between dietary fat quality and T2DM can dissolve complexities and take the interrelations of dietary fats into consideration. Our study also had some limitations. First, the sample size of the study and follow-up duration were small compared with other similar studies. Second, there was a risk of bias due to the large number of excluded participants who did not complete the FFQ at baseline; however, it was shown that there was no significant difference between the characteristics of participants who did not complete the FFQ and those of the total population in the third phase of TLGS^(25)^. Third, we used a self-reported FFQ, so measurement error was not excluded. Fourth, due to the limited data of the Iranian Food Composition Table on the nutrient content of raw foods and beverages, we used the USDA food composition table, which may affect the results. Fifth, we did not have enough information to realise the actual date of diagnosis of pre-diabetes or T2DM. Finally, although we adjusted for a wide range of potential confounders, we were unable to evaluate the impact of genetic factors and environmental risk factors, such as air pollution, neighbourhood walkability, green space and climate condition.

### Conclusion

In conclusion, this study revealed that there is no significant association between FQI and risk of pre-diabetes and T2DM. Further prospective and clinical trial studies are needed to clarify the association between dietary fat quality and T2DM and pre-diabetes.
